# Genomic insights into potential interdependencies in microbial hydrocarbon and nutrient cycling in hydrothermal sediments

**DOI:** 10.1186/s40168-017-0322-2

**Published:** 2017-08-23

**Authors:** Nina Dombrowski, Kiley W. Seitz, Andreas P. Teske, Brett J. Baker

**Affiliations:** 1 Department of Marine Science, Marine Science Institute, University of Texas Austin, Port Aransas, TX USA; 2 Department of Marine Sciences, University of North Carolina at Chapel Hill, Chapel Hill, NC USA

**Keywords:** Deep sea, Microbiome, Metagenome, Bacteria, Archaea, Population genomes, Comparative genomics

## Abstract

**Background:**

Deep-sea hydrothermal vents are hotspots for productivity and biodiversity. Thermal pyrolysis and circulation produce fluids rich in hydrocarbons and reduced compounds that stimulate microbial activity in surrounding sediments. Several studies have characterized the diversity of Guaymas Basin (Gulf of California) sediment-inhabiting microorganisms; however, many of the identified taxa lack cultures or genomic representations. Here, we resolved the metabolic potential and community-level interactions of these diverse communities by reconstructing and analyzing microbial genomes from metagenomic sequencing data.

**Results:**

We reconstructed 115 microbial metagenome-assembled genomes comprising 27 distinct archaeal and bacterial phyla. The archaea included members of the DPANN and TACK superphyla, Bathyarchaeota, novel *Methanosarcinales* (GoM-Arc1), and anaerobic methane-oxidizing lineages (ANME-1). Among the bacterial phyla, members of the *Bacteroidetes*, *Chloroflexi*, and *Deltaproteobacteria* were metabolically versatile and harbored potential pathways for hydrocarbon and lipid degradation and a variety of respiratory processes. Genes encoding enzymes that activate anaerobic hydrocarbons for degradation were detected in *Bacteroidetes*, *Chloroflexi*, Latescibacteria, and KSB1 phyla, while the reconstructed genomes for most candidate bacteria phyla (Aminicenantes, Atribacteria, Omnitrophica, and Stahlbacteria) indicated a fermentative metabolism. Newly obtained GoM-Arc1 archaeal genomes encoded novel pathways for short-chain hydrocarbon oxidation by alkyl-coenzyme M formation. We propose metabolic linkages among different functional groups, such as fermentative community members sharing substrate-level interdependencies with sulfur- and nitrogen-cycling microbes.

**Conclusions:**

Overall, inferring the physiologies of archaea and bacteria from metagenome-assembled genomes in hydrothermal deep-sea sediments has revealed potential mechanisms of carbon cycling in deep-sea sediments. Our results further suggest a network of biogeochemical interdependencies in organic matter utilization, hydrocarbon degradation, and respiratory sulfur cycling among deep-sea-inhabiting microbial communities.

**Electronic supplementary material:**

The online version of this article (doi:10.1186/s40168-017-0322-2) contains supplementary material, which is available to authorized users.

## Background

Marine sediments form the largest repository of organic carbon and the most extensive habitat for microbial life, where sediment-inhabiting microorganisms drive nutrient and carbon cycling [1–4]. Guaymas Basin (GB), a hydrothermally active seafloor-spreading center in the Gulf of California, is characterized by high primary production, rapid sedimentation and deposition, and hydrothermal processing of buried organic matter within its massive sediment cover [5, 6]. Hydrothermal alterations transform the deposited carbon and produce large amounts of methane, petroleum-like compounds (alkanes and polycyclic aromatic hydrocarbons (PAHs)), organic acids, and ammonia [5, 7–9]. These substrates get distributed throughout the sediments by hydrothermal circulation and are readily assimilated by the local microbial community [10]. For example, hydrothermal fluids containing high concentrations of methane (> 15 mM) mixing with seawater sulfate (28 mM) favor the anaerobic oxidation of methane (AOM), which occurs at high temperatures in Guaymas Basin sediments [11–13]. AOM is usually carried out in a syntrophic relationship between anaerobic methanotrophic (ANME) archaea and members of the *Deltaproteobacteria* that couple the oxidation of methane with the reduction of sulfate [14]. In hydrothermal sediments of Guaymas Basin, AOM is also performed at high temperatures by a syntrophic consortium of ANME-1 archaea and the deeply-branching, hydrogenotrophic sulfate-reducing bacterium *Candidatus* Desulfofervidus auxilii [15–17]. In contrast to this well-studied interaction, little is known about the degradation of other abundant hydrocarbons (such as PAHs and alkanes) by the GB microbiome.

The microbial community composition of GB sediments has been thoroughly described using marker-gene studies [12, 13]. For example, an automated ribosomal spacer analysis indicated that > 80% of the detected operational taxonomic units (OTUs) were shared across different temperature and depth profiles, suggesting a high connectivity across sediments [12, 18]. Furthermore, depth and temperature regime are thought to influence community assembly [18]. Temperatures in GB sediments range from ~ 3 °C at the surface up to ~ 200 °C at 30- to 50-cm depth, which is accompanied with geochemical zonation due to mixing of hot vent fluids with cold ocean water [12, 18, 19]. 16S rRNA gene sequencing of Guaymas Basin sediments has revealed numerous bacterial and archaeal lineages [12, 13]. Among the archaea, ANME-1 members are frequently detected, as well as other common deep-sea lineages including Marine Benthic Group D (MBG-D) and Bathyarchaeota (formerly MCG). Consistently detected bacterial community members include *Epsilonproteobacteria*, *Deltaproteobacteria*, such as the uncultured SEEP-SRB2, *Candidatus* Desulfofervidus auxilii (previously HotSeep-1) lineages, *Bacteroidetes*, or *Chloroflexi* [12, 13].

Despite our knowledge about the geochemistry of GB sediments, and the taxonomic composition of its microbial communities, single-gene studies lack insights into the metabolic capacities and ecological connectivity in this unique deep-sea environment. To address this gap, we obtained metagenomic libraries from two GB sediment sites. Accompanying biogeochemical data for these two sites suggest that in addition to abundant methane, short-chain alkanes (C_1_ to C_6_) are available in substantial concentrations (20 to 100 μM) [13]. Consistent with the frequent detection of sulfate-reducing microbial populations that use hydrogen as preferred electron donor, porewater hydrogen concentrations are consistently low (< 10 nM) [20]. Therefore, we hypothesize that this habitat is suitable for syntrophic alkane oxidation by consortia of alkane-oxidizing archaea and sulfate-reducing bacteria that catalyze the terminal electron transfer from alkane to sulfate. Additionally, we aim to address whether the deposition and subsequent alteration of abundant organic carbon favors an unusually diverse microbial community in Guaymas Basin sediments [5, 6]. From the two GB sediment sites, we were able to reconstruct 115 metagenome-assembled genomes (MAGs) belonging to 27 bacterial and archaeal phyla, allowing us to begin to resolve the metabolic potential of a variety of uncultured community members. Physiological analyses of these communities revealed novel pathways for hydrocarbon processing and potential ecological interdependencies.

## Results

### Genome reconstructions and community composition

We obtained ~ 242 gigabases of Illumina shotgun sequencing data from two GB hydrothermal sediment sites, where sediments cores were collected during *Alvin* dives 4484 and 4572 in December 2008 and 2009, respectively (Additional file 1: Tables S1 and S2, details in the “Methods” section). Samples from two depth profiles from each of these two representative sites were selected for sequencing based on the availability of accompanying biogeochemical data, thermal gradients, and evidence of a diverse microbial community [12, 13, 21]. De novo assembly and tetranucleotide (and coverage) binning of sequencing data from these four samples allowed the reconstruction of 38 archaeal and 77 bacterial draft metagenome-assembled genomes (MAGs, completeness > 50%, Additional file 1: Table S3, Figure S1). Of those MAGs, 51 were estimated to be > 70% and 20 to be > 80% complete, with minimal single-gene duplications (≤ 10%, Additional file 1: Table S3).

**Fig. 1:**
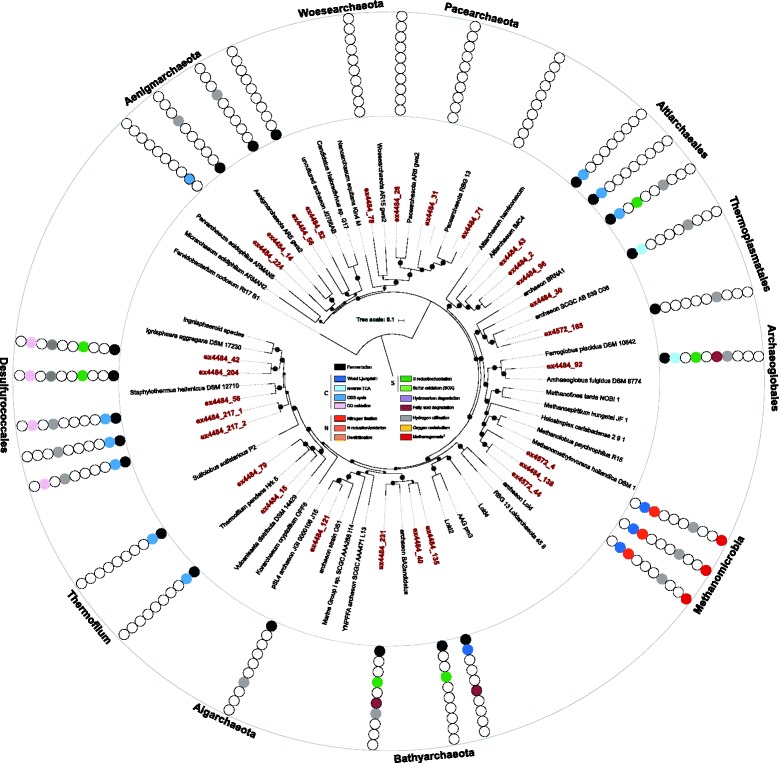
Phylogenetic and metabolic representation of assembled archaeal MAGs. Maximum-likelihood-based phylogenetic tree of up to 15 concatenated ribosomal proteins (rpL2, 3, 4, 5, 6, 14, 15, 18, 22, 24 and rpS3, 8, 10, 17, 19) from archaeal MAGs assembled from Guaymas Basin deep-sea sediments. MAGs were assembled from dive 4484 and 4572 (*dark red*). Only includes MAGs with ≥ 8 ribosomal proteins. *Circles* represent bootstrap values > 70% (bootstrap values were generated using the ultrafast bootstrap method with 1000 replications). Core metabolic processes: metabolic reconstruction was based on gene calling and annotation using IMG/MER, RAST, a custom blast and hmmer database search (see the “Methods” section). ^1^The “Methanogenesis” identifier reflects the presence of *mcrA* and related genes identified as key components of methanogenesis and the oxidation of methane and butane through reverse methanogenesis (Additional file 1: Table S4)

**Fig. 2:**
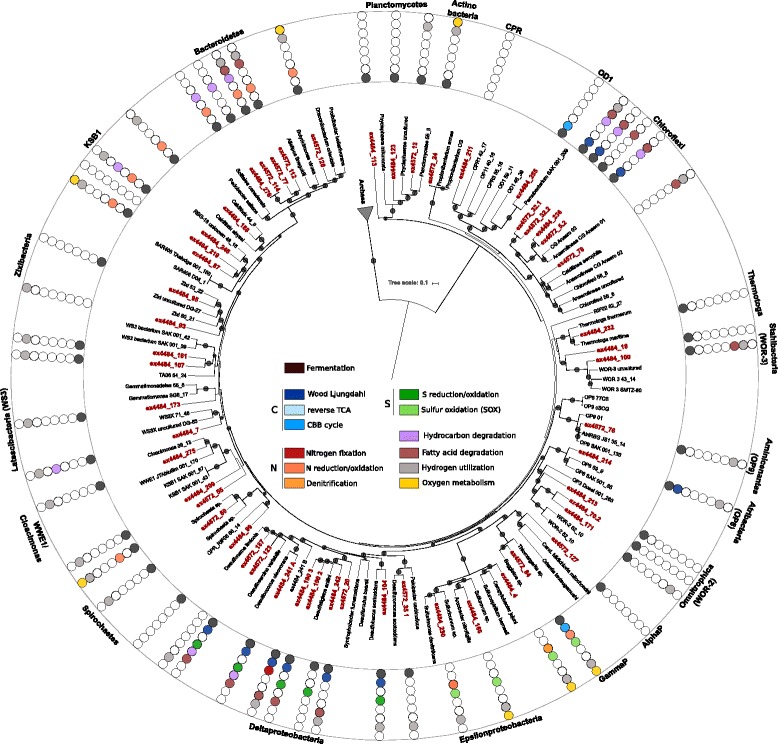
Phylogenetic and metabolic representation of assembled bacterial MAGs. Maximum-likelihood-based phylogenetic tree of up to 15 concatenated ribosomal proteins (rpL2, 3, 4, 5, 6, 14, 15, 18, 22, 24 and rpS3, 8, 10, 17, 19) from bacterial MAGs assembled from Guaymas Basin deep-sea sediments. MAGs were assembled from dive 4484 and 4572 (*dark red*). Only includes MAGs with ≥ 8 ribosomal proteins*. Circles* represent bootstrap values > 70% (bootstrap values were generated using the ultrafast bootstrap method with 1000 replications). Core metabolic processes: metabolic reconstruction was based on gene calling and annotation using IMG/MER, RAST, a custom blast and hmmer database search (see the “Methods” section). AlphaP: *Alphaproteobacteria*, GammaP: *Gammaproteobacteria*

The reconstructed MAGs comprise a total of 9 archaeal and 18 bacterial phyla (Figs. 1 and 2, Additional file 1: Figures S2–S7, Table S3). Overall, this community is taxonomically diverse and includes lineages of the Bathyarchaeota, *Methanosarcinales* (novel GoM-Arc1), *Methanomicrobia* (ANME-1 groups), *Thermoplasmatales* (uncultured VC2.1 Arc6, CCA47 lineages), *Bacteroidetes*, *Chloroflexi*, *Deltaproteobacteria*, and *Gammaproteobacteria*. Additionally, we identified several MAGs from a variety of little understood candidate phyla: Aminicenantes (OP8), Atribacteria (OP9), Cloacimonas, Omnitrophica (WOR-2), Latescibacteria (WS3), WOR-3, Zixibacteria, as well as Pacearchaeota and Geothermarchaeota. Phylum WOR-3 MAGs were first recovered from estuary sediments and have also been seen in groundwater sediments [22, 23]; we propose they be named Candidatus “Stahlbacteria” after Dr. David Stahl, an accomplished environmental microbiologist and early proponent of 16S rRNA-based microbial ecology. The placement of Candidatus Stahlbacteria as a new phylum was supported both by a phylogenetic analysis using 16 concatenated ribosomal marker genes as well as an extended marker set using 37 concatenated genes for a more robust phylogenetic placement (see the “Methods” section; Fig. 2, Additional file 1: Figure S7). The most abundant MAGs at both sites were assigned to a gammproteobacterium of the family *Beggiatoaceae* (ex4572_84) and an archaeum of the phylum *Euryarchaeota* of the class *Methanomicrobia* (ex4572_4) (Additional file 1: Figures S8, S9). We did detect a few site-specific differences, such as the presence of Omnitrophica in samples from dive 4484 but not 4572, suggesting there are community-level differences across sample locations and/or years (Additional file 1: Figures S8, S9). However, to further resolve potential site-/time-dependent genome-level differences, a broader sampling scale would be required.

### Carbon metabolism

To infer the metabolic potential of GB community members, we annotated genes within each of the MAGs using a variety of protein databases (see the “Methods” section). Functional interpretations were aided by linking individual genes to complete pathways within each MAG. Notably, the basic features of inferred physiologies were fairly consistent within organisms of the same phyla, allowing for MAGs to be grouped for metabolic comparisons (Figs. 1 and 2, Additional file 1: Table S4).

**Fig. 3:**
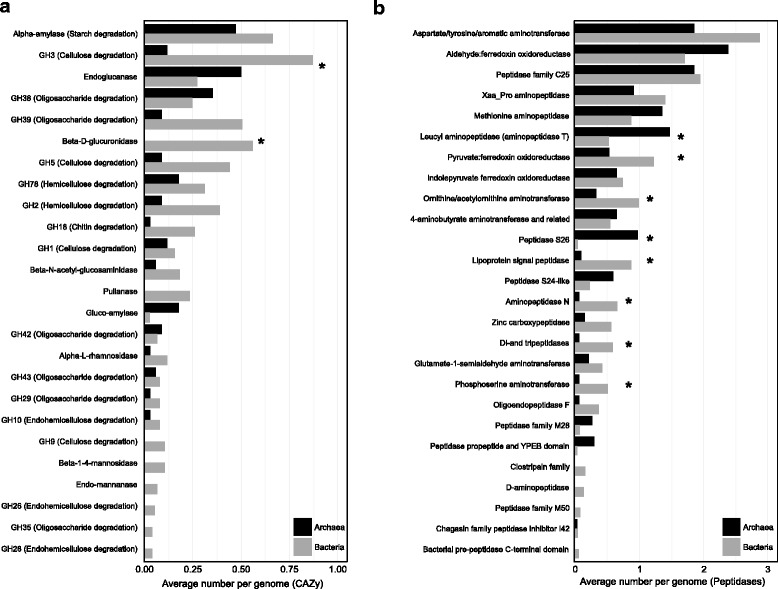
Relative abundance of genes encoding for carbohydrate-degrading enzymes and peptidases among archaeal and bacterial MAGs. **a** Relative abundance of carbohydrate-degrading enzymes (CAZy) and **b** peptidases among archaeal (*black*, *n* = 34) and bacterial (*gray*, *n* = 77) MAGs. *: *P*-value <0.05 (non-parametric Mann–Whitney test, Bonferroni corrected). Normalized to the total number of archaeal and bacterial MAGs

**Fig. 4:**
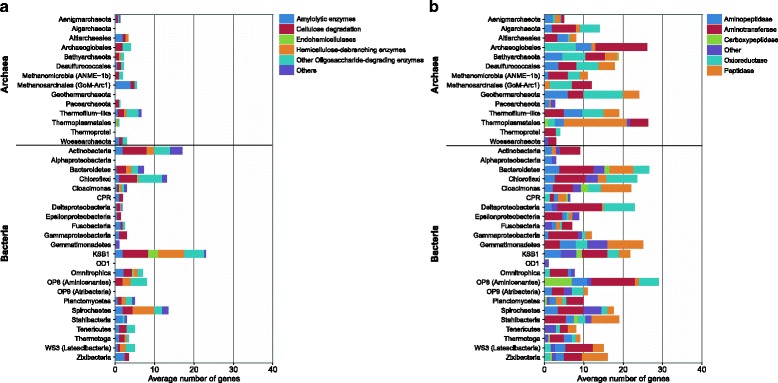
Relative abundance of genes encoding for carbohydrate-degrading enzymes and peptidases among archaeal and bacterial phyla. **a** Relative abundance of carbohydrate-degrading enzymes (CAZy). **b** Peptidases encoded in archaeal and bacterial phyla (average number of genes per phylum)

Pathways involved in the degradation of detrital organic matter (including complex carbohydrates, lipids, and proteins) were prevalent across both archaeal and bacterial MAGs (Fig. 3). The most numerous carbohydrate-degradation genes encoded for alpha-amylase (starch degradation), members of the glycoside hydrolase family 3 (GH3; cellulose degradation), and endoglucanase (cellulose degradation). Several carbohydrate-degradation genes that were abundant in bacteria, including GH3, beta-D-glucoronidase, pullanase, and beta-1,4-mannosidase, were rare or even absent from archaea (Additional file 1: Table S5, *P* value < 0.05 based on a non-parametric Mann–Whitney test, Bonferroni corrected). This finding suggests that bacteria have access to a more diverse carbohydrate pool. *Chloroflexi*, KSB1, *Spirochaetes*, *Methanosarcinales*, and *Thermofilum* contained the highest number of carbohydrate-degrading genes, while no clear lineage-specific pattern was observed for peptidases (Fig. 4, Additional file 1: Figure S10). Aside from carbohydrate-degradation, the ability to degrade lipids via the beta-oxidation pathway was common to members of *Bacteroidetes*, *Chloroflexi*, and *Deltaproteobacteria*. Additionally, we identified the key beta-oxidation genes for acyl-CoA dehydrogenase, enoyl-CoA hydratase, 3-hydroxyacyl-CoA dehydrogenase, and acetyl-CoA C-acyltransferase in Geothermarchaeota, *Archaeoglobales*, and the majority of the Bathyarchaeota genomes.

The ability to ferment (via glycolysis) appeared to be almost universal among GB archaea and bacteria (Figs. 1 and 2, Additional file 1: Table S4). Bacteria likely ferment various carbon sources, including complex carbohydrates (cellulose, hydrocarbons) and peptides (after hydrolysis into monomeric sugars and amino acids) predominantly into hydrogen and ethanol, followed by lactate and acetate. Fe,Fe-hydrogenases for producing H_2_ were identified in *Bacteroidetes*, *Fusobacteria*, and Omnitrophica [24]. The archaea identified are primarily predicted to produce hydrogen and acetate, and none appear to be able to produce lactate. Among the archaea, Ni,Fe-hydrogenases were found in the majority of phyla and orders, including Aenigmarchaeota, *Desulfurococcales*, and *Thermoplasmatales*, while Fe,Fe-hydrogenases were found in various bins but could not be consistently linked to specific lineages.

### Hydrocarbon utilization

Short-chain (petroleum-like) C_2_-C_10_ hydrocarbons are rapidly generated by hydrothermal pyrolysis and thus are a particularly abundant source of carbon and energy in hot GB sediments [5]. No genes for the aerobic degradation of these hydrocarbons were identified in the GB MAGs, but genes for anaerobic hydrocarbon degradation were detected among several phyla, including *Bacteroidetes*, *Chloroflexi*, *Deltaproteobacteria*, and the candidate divisions Latescibacteria (WS3), and KSB1 (Fig. 2, Additional file 1: Table S4). *Deltaproteobacteria*, *Chloroflexi*, Latescibacteria, and KSB1 contain genes that potentially encode for the benzylsuccinate synthase (*bssA*) and alkylsuccinate synthase (*assA*), which can activate PAHs or alkanes using the fumarate addition mechanism [25–27].

**Fig. 5:**
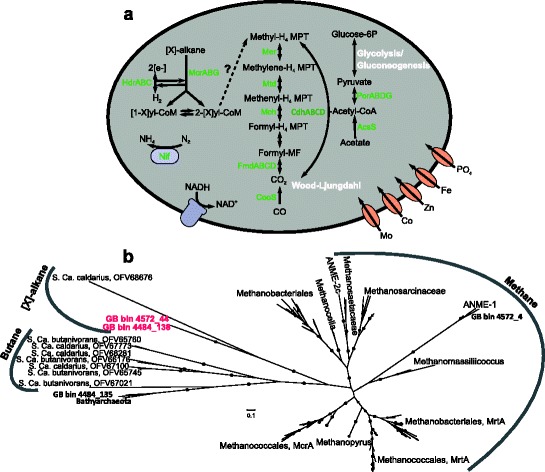
Physiological capabilities of GoM-Arc1 archaea recovered from the GB sediments. **a** Diagram of the functional genes and metabolic pathways found in the GoM-Arc1 archaeal MAGs recovered from GB. The proteins encoded by both of the MAGs are shown in *green*. **b** Phylogenetic tree of McrA proteins recovered from the GB sediment genomes. Sequences found in GB genomes are highlighted in *red*. The phylogeny was generated using RAxML methods, and *circles* represent bootstrap values > 90%. *S. Ca.* *Candidatus* Syntrophoarchaeum

The coexistence of high methane (up to 15 mM) and pore-water sulfate (up to 28 mM) concentrations in the well-ventilated Guaymas sediments create favorable conditions for the sulfate-dependent, anaerobic oxidation of methane (AOM; Additional file 1: Table S1) [12, 13]. We recovered a *Methanomicrobia* MAG (bin 4572_4) belonging to the archaeal ANME-1 lineage, which are known to be involved in syntrophic anaerobic methane oxidation (ANME; Additional file 1: Figure S2) [28]. As previously described for other ANME members, this MAG encodes a complete pathway for methane oxidation via reverse methanogenesis (Fig. 5a) [29]. The key gene of this pathway is the *mcrA* gene, which encodes for the methyl–coenzyme M reductase that cleaves methane to form methyl coenzyme M [30]. This organism’s predicted McrA is closely related to previously described ANME-1 proteins (Fig. 5b). We did not detect any genes involved in electron transfer to an external electron acceptor within the *Methanomicrobia* MAGs; therefore, these archaea likely require a syntrophic interaction with bacterial sulfate reducers. These sulfate reducers characteristically belong to the *Desulfosarcina*/*Desulfococcus* (DSS) or *Desulfobulbus*-related (DSB) lineage of the *Deltaproteobacteria* [31, 32]. We successfully reconstructed two sulfate-reducing MAGs assigned to the DSS lineage that could be potential syntrophic partners of ANME-1 (Fig. 2, Additional file 1: Figure S3, Table S3).

Aside from the *mcrA* gene identified in ANME-1, we detected further genes in the Bathyarchaeota (bin ex4484_135) and the first representative MAGs of the GoM-Arc1 group (bins ex4572_44 and ex4484_138; Fig. 5, Additional file 1: Figure S2, Table S4) [28]. Phylogenetic analyses of these proteins revealed they are considerably divergent from those of known methane oxidizers and methanogens (Fig. 5b). In addition to having a methyl-coenzyme M reductase (subunits ABG), these archaea also contain genes encoding for enzymes required in other steps of anaerobic butane oxidation including heterodisulfide reductases (HdrABC), tetrahydromethanopterin S-methyltransferase (MtrABCDEFGH), methylenetetrahydromethanopterin reductase (Mer), N^10^-methenyl-H_4_MPT cyclohydrolase (Mch), formylmethanofuran dehydrogenases (FmdABCD), and a complete Wood-Ljungdahl pathway (Fig. 5a). However, neither of the GoM-Arc1 genomes contains genes encoding for the butyryl-CoA oxidation or beta-oxidation pathway, and the *mcrA* genes belonging to GoM-Arc1 are phylogenetically distinct from those previously shown to be involved in butane oxidation in *Candidatus* Syntrophoarchaeum spp. [33]. It has been shown that the anaerobic oxidation of hydrocarbons can be achieved by a syntrophic interaction with sulfate-reducing bacteria [33]. We obtained 15 *Deltaproteobacteria* MAGs belonging to a variety of taxa. The MAGs belonging to the family *Desulfobacteraceae* (bins ex472_123 and ex4572_130) contain genes that encode a type IV pilus (PilA) and extracellular cytochromes, which have been implicated in transferring electrons between archaea and bacteria [34, 35].

### Community interactions

**Fig. 6:**
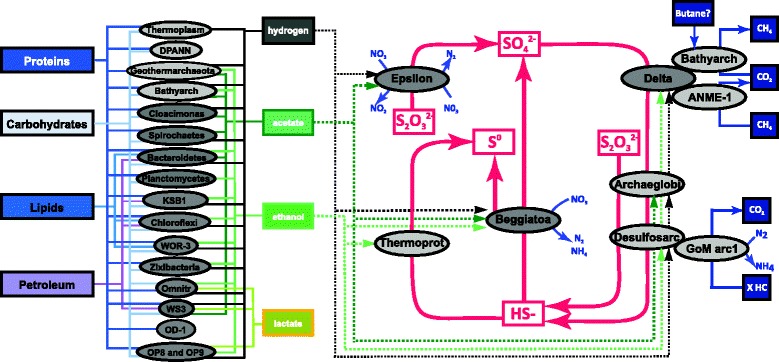
Interactions among carbohydrate utilization, fermentation, and respiratory pathways. *Arrows* represent metabolic capabilities that were identified in the MAGs reconstructed from Guaymas Basin deep-sea sediments based on gene calling and annotation using IMG/MER, RAST, a custom blast and hmmer database search. The *dashed lines* on the right represent potential electron donors for the anaerobic respiration processes. OP8: Aminicenantes, OP9: Atribacteria, Omnitr: Omnitrophica, WOR-3: Candidatus Stahlbacteria, WS3: Latescibacteria

To investigate other potential biogeochemical interdependencies in the GB sedimentary communities, we mapped the ecological roles of all the microbes that were obtained (Fig. 6). This revealed several potential substrate-dependent interactions among fermentative community members and sulfur- and nitrogen-cycling organisms. Sulfate reduction pathways are encoded in *Deltaproteobacteria* and *Archaeoglobales* archaeal MAGs (Figs. 1 and 2, Additional file 1: Table S4). Furthermore, the *Desulfurococcales* within the archaea are predicted to be capable of S^0^ reduction and Bathyarchaeota MAGs encode the alpha and beta subunits of the anaerobic sulfite reductase (asrAB). Hydrothermal fluids also provide millimolar concentrations of sulfide in GB sediments [12, 13, 19]. This sulfide could be oxidized by the dominant bacterium affiliated with the *Beggiatoaceae* (bin ex4572_84) [36], which contains genes encoding sulfide quinone reductases (*sqr*), sulfur oxidases (*soxBY*), and thiosulfate reductase (*phsA*). The *Epsilonproteobacteria* are a second phylogenetic lineage likely involved in sulfur oxidation. Based on the presence of sox genes (subunits *soxBCY*) in the reconstructed epsilonproteobacterial genomes (bins ex4484_166, ex4484_230, ex4484_65 and ex4484_4), we propose they are primarily involved in intermediate sulfur (thiosulfate) cycling.

The *Beggiatoaceae* and *Epsilonproteobacteria* appear to be capable of chemoautotrophic coupling of nitrate reduction to sulfur oxidation, consistent with previous genomic and physiological analyses [19, 36, 37]. Nitrate reduction extends beyond the *Proteobacteria*; many other bacteria belonging to *Bacteroidetes*, KSB1 and WWE1 encode nitrate reductases (*napA*) and likely require organic electron donors. In addition to utilizing hydrogen as electron donor, these organisms are predicted to take up fermentation byproducts and presumably oxidize them by denitrification; ethanol can potentially be used by *Chloroflexi* and KSB1, and acetate by *Bacteroidetes* and WWE1. Hydrogenases were often detected in nitrate reducers (Figs. 1 and 2). We found genes encoding for Ni,Fe-Hydrogenases in *Epsilonproteobacteria,* suggesting they are able to oxidize sulfur and H_2_, likely coupled to the reduction of nitrate. Furthermore, *Deltaproteobacteria* are predicted to couple sulfate reduction with the oxidation of carbohydrates, fatty acids, or petroleum-like compounds. Overall, these findings highlight the multitude of substrate-level dependencies driving nutrient cycles in GB sediments.

## Discussion

We assembled MAGs of 115 GB-inhabiting microbes, comprising 9 archaeal and 18 bacterial phyla, many of which are abundant in GB sediments based on diversity surveys from the same sediments [13]. The most dominant archaeal and bacterial community members belonged to a member of the archaeal ANME-1 group and the *Beggiatoaceae*, respectively (Additional file 1: Figures S8, S9). The high abundance of both taxa is expected, as both sediment locations are covered by dense *Beggiatoaceae* mats, and *Methanosarcinales* have been previously defined as abundant community members [13, 19, 36].

The reconstruction of MAGs sheds light on the metabolic potential of individual community members. Genes for the degradation of organic matter and the ability to ferment appeared to be nearly universal across GB archaea and bacteria (Figs. 1 and 2). Overall, GB microbes contained multiple genes to degrade and assimilate a broad range of substrates, including carbohydrates, peptides, petroleum compounds, and fatty acids, indicating that the utilization of varied types of sedimentary organic carbon –fresh photosynthetic biomass as well as fossil hydrocarbons - is a central function of microbial communities in these sediments. This broad access to different carbon sources appears highly characteristic for sediment environments, including aquifers and estuaries, suggesting that the microbial utilization of organic carbon is a central driver of nutrient cycles in marine sediments [22, 23]. Although Guaymas Basin has unique hydrothermally active sediments, these sediments share many characteristics with other sedimentary environments, such as the White Oak River estuary [38, 39]. Both sediment environments are rich in photosynthetically derived organic matter, sulfide and methane, which potentially explain similarities among these different habitats.

A unique feature of GB sediments is the high abundance of short-chain (petroleum-like) C_2_-C_10_ hydrocarbons that are generated during hydrothermal pyrolysis [5]. Interestingly, we identified several potential enzymes for anaerobic hydrocarbon degradation, including putative activating enzymes involved in PAH and alkane degradation in *Bacteroidetes, Chloroflexi*, *Deltaproteobacteria*, and candidate phyla Latescibacteria and KSB1 (Fig. 2). *Deltaproteobacteria* are often enriched in oil-contaminated sediments and can couple the oxidation of hydrocarbons with the reduction of sulfate [40, 41]. However, to our knowledge, this is the first documentation of genes predicted to be involved in anaerobic hydrocarbon degradation in the candidate phyla Latescibacteria and KSB1. The widespread distribution of genes for hydrocarbon degradation beyond members of the *Proteobacteria* extends the potential metabolic diversity found in deep-sea sediments and calls for hydrocarbon degradation studies of cultivable *Chloroflexi* and *Bacteroidetes* in Guaymas Basin.

Across the archaeal genomes, ANME-1, Bathyarchaeota and Gom-Arc1 members are able to oxidize methane and other short-chain alkanes using McrA (Fig. 5). The ANME-1 McrA protein is likely involved in AOM that is coupled with sulfate reduction in *Deltaproteobacteria* [28]. For example, it has been shown that ANME-1 archaea and members of the *Desulfosarcina* (family *Desulfobacteraceae*) are closely associated in sediments [42]. We reconstructed two MAGs that are related to the *Desulfobacteraceae* and are able to reduce sulfate and encode for potential electron carriers (Fig. 2, Additional file 1: Figure S3). Therefore, we hypothesize that the ANME-1 are coupling AOM with sulfur reduction carried out in a syntrophic interaction with these *Desulfobacteraceae*. This attribution has to be validated since members of this family also thrive as free-living sulfate reducers in marine sediments. At least in the case of ANME-2 archaea, the *Desulfosarcina* syntrophs are members of a specific 16S rRNA gene-defined clade, the SRB1a cluster [31]; since we could not retrieve 16S rRNA genes of these MAGs, further experiments are required to confirm this taxonomic affiliation. The Bathyarchaeota type *mrcA* is related to those recently described in genomes of this group from a deep aquifer that have been shown to be able to oxidize butane instead of methane [43]. The two GoM-arc1 archaeal MAGs contained novel alkyl-coenzyme M reductase (*mcr*) genes that clustered apart from both the ANME-1 and bathyarchaeotal *mcrA* genes. Based on the phylogeny of the *mcrA* gene and the absence of the beta-oxidation pathway [33], we conclude that the GoM-Arc1 archaea are not capable of oxidizing butane, but rather another type of short-chain hydrocarbon, using a novel pathway to feed the McrA-activated hydrocarbon into the reverse methanogenesis pathway. Overall, the phylogenetic comparison of these McrA proteins suggests that archaea use them for a broader repertoire of hydrocarbon substrates than has been realized.

Elucidating the metabolisms of individual community members allowed us to map potential biogeochemical interdependencies among the members of this hydrothermal sediment community. The fermentative organisms might metabolize complex carbon sources and thereby provide substrates to fuel the anaerobic respiration of both nitrate and sulfate. The prevalence of sulfate-dependent pathways is consistent with the high pore-water sulfate concentrations in GB sediments; from several millimolar up to full seawater strength (28 mM) [12, 13]. Notably, we found likely syntrophic relationships among both fermenting and respiring organisms, aside from the well-described interaction of ANME archaea with *Deltaproteobacteria* [31]. For example, the sulfur cycle was fragmented across several community members, suggesting that biogeochemical nutrient cycles are partitioned among individual community members [23]. Future experiments, including gene expression studies or amino acid tagging techniques, are needed to confirm the activity and interconnectivity of Guaymas Basin sediment-inhabiting microbes. Additionally, selecting a wider dataset, including sites spanning a broader depth and temperature profile, will help determine whether the functional diversity in this study reflects the diversity present in hydrothermal vents as a whole.

## Conclusions

GB hydrothermal vent sediments are hotspots for microbial carbon cycling and contain high concentrations of methane and hydrocarbons, including alkanes and PAHs [5, 12, 13]. The metabolic reconstruction of 115 new microbial MAGs revealed the substrate-dependent connectivity among deep-sea inhabiting microorganisms. Mapping of the inferred ecological roles of all these organisms indicated potential biogeochemical interdependencies in organic matter utilization, hydrocarbon degradation, and respiratory sulfur and nitrogen cycling. Of particulate interest is the identification of potentially novel enzymes for hydrocarbon degradation in *Chloroflexi*, *Bacteroidetes*, and candidate phyla Latescibacteria and KSB1. Additionally, the first genomes of the GoM-arc1 archaea contained novel alkyl-coenzyme M (*mcr*) genes and pathways for the oxidation of an unknown short-chain alkane. These findings extend the spectrum of hydrocarbon-degrading physiologies among deep-sea inhabiting microorganisms and call for hydrocarbon degradation studies among cultivable microbes in Guaymas Basin.

## Methods

### Sampling

Guaymas Basin sediment samples were collected from the Gulf of California (27° N 0.388, 111° W 24.560) at a depth of approximately 2000 m below the water surface. Sediment cores were sampled during two Alvin dives (dive 4484 core #1, December 6, 2008, and dive 4572 core #18, December 3, 2009) from a hydrothermal mat area at the base of Mat Mound and from a 200-m distant hydrothermal area, termed Marker 27 (Additional file 1: Table S1). Dense mats of *Gammaproteobacteria* of the family *Beggiatoaceae* covered both sites, with a white mat dominating at site 4484 and an orange mat at site 4572. Intact sediments from both dives were collected using polycarbonate cores (45 cm in length, 6.25-cm interior diameter), subsampled into centimeter layers under N_2_ gas in the ship’s laboratory and immediately frozen at − 80 °C. Sediment subsamples for DNA isolation were taken from depths 0–1 and 3–4 cm in core 4484-1 and 0–3 and 12–15 cm in core 4572-18. Metadata for dive 4484 and 4572, including details on the geochemistry (i.e., methane concentrations, dissolved organic carbon concentrations, sulfate and sulfide concentrations) as well as thermal profiles of the sampling site, are available to compare microbial community composition across sediment cores [12, 19, 21]. For 4484-1, full metadata are included in Dowell et al. 2016. For 4572-18, full metadata are included in McKay et al. 2016. Pictures of the sampling locations are included in Teske et al. 2016.

### Metagenomic sequencing

Total DNA from 10 g of sediment from each of the four samples (see above) was extracted using the MoBio PowerMax soil kit. DNA concentrations were measured using a Qubit™ 3.0 Fluorometer, and a final concentration of 10 ng/μl of each sample (using a total amount of 100 ng) was used to prepare libraries for paired-end Illumina (HiSeq 2500) sequencing. Illumina library preparation and sequencing was performed by the GSAF (Genome Sequencing and Analysis Facility) at the University of Texas at Austin. Sequencing was performed on an Illumina HiSeq 2500 with the following specifications: high throughput run mode, run type paired end 2 × 125 bp, 6 × 4.0E8 target reads (millions), insert size approximately 360–420 bp and ~ 5% PhiX control spike-in. This sequencing approach provided a total of ~ 242 gigabases of sequencing data (411,732,022/520,750,012/552,634,962/452,269,130 reads from the sediment samples 4484 0–1 cm/4484 3–4 cm/4572 0–3 cm/4572 12–15 cm, respectively).

Raw Illumina shotgun genomic reads were separated from Illumina artifacts by removing the adaptors and DNA spike-ins from the forward and reverse reads. Reads with an average quality score < Q20 and a read length < 50 bps were removed using cutadapt [44]. Afterwards, reads were interleaved using interleave_fasta.py and the interleaved sequences were trimmed using Sickle with default settings [45]. The script for interleave_fasta.py can be found at https://github.com/jorvis/biocode/blob/master/fasta/interleave_fasta.py. Metagenomic reads from dive 4484 and 4572 (concatenated per depth profile for better coverage) were individually assembled using IDBA-UD using the following parameters: --pre_correction, -mink 75, -maxk 105, --step 10, --seed_kmer 55 [46]. This yielded a total of 3,139,208 and 159,5687 scaffolds from sample 4484 and 4572, respectively, including scaffolds with a minimum and maximum scaffold length ranging from 200 to 177,401 bp (maximum scaffold length for sample 4484 and 4572: 177,401 and 133,414, respectively).

Metagenomic binning was performed on assembled samples from dive 4484 and 4572 by calculating tetranucleotide frequencies of scaffolds with a minimum length of 5000 bp (including a total of 58,488 scaffolds with a total length of 591,330,171 bp) [47]. The resulting Emerging Self-Organizing Maps (ESOM) were manually sorted and curated (Additional file 1: Figure S1) [47]. Metagenomic binning was enhanced by incorporating reference genomes as genetic signatures for the assembled contigs into ESOM [47, 48]. Thereby, we assembled 77 bacterial and 38 archaeal metagenomic assembled genomes (MAGs) with a completeness above 50%. After binning, MAGs were linked to the original sediment samples based on their unique scaffold ID. CheckM was employed to evaluate the accuracy of the binning approach by determining the percentage of completeness and contamination (Additional file 1: Table S3) [49]. Contaminants that were identified based on their phylogenetic placement (wrong taxonomic assignment compared to the average taxonomic assignment of the genes assigned to each bin), GC content (> 25% difference compared to the mean of all scaffolds assigned to each bin), or confidence level (> 25% differences compared to the mean of all scaffolds assigned to each bin) were manually removed from each MAGs.

### Genome coverage

To determine the relative abundance of each MAG across the four sequenced Guaymas Basin sediment samples, we mapped scaffolds from all MAGs against the original metagenomic sequencing reads using BWA using default settings [50]. To detect exact matches to the original sequencing data, we only considered matches where the complete sequence of the raw sequence read (125 bp) matched the MAG. The relative abundance of each MAG was calculated by normalizing the recruited reads per MAG by the genome size, accounting for differences in sampling depth of the respective metagenome and then multiplying by 1,000,000. A total of 31,363,251 archaeal and 116,888,222 bacterial reads mapped back to the original metagenomes and represented ~ 15% (archaea) and ~ 49% (bacteria) of the sequenced community.

### Gene calling, taxonomic assignment, and functional characterization

Gene calling and taxonomic assignment for the four metagenomic samples and individual MAGs was performed using the Joint Genome Institute-Production Genomics Facility (JGI-PGF) integrated microbial genomes with microbiome (IMG/M) system. The IMG output was linked to the MAGs by their unique scaffold ID, which was also used to extract protein sequence information for further analyses. Additionally, gene calling for individual MAGs was performed using RAST (Rapid Annotation using Subsystem Technology) [51, 52]. For RAST, individual MAGs were uploaded using the Network-Based SEED API using the command svr_submit_RAST_job, selecting the RAST gene caller method.

For a further functional characterization, individual MAGs were analyzed using the IMG/M systems output, the SEED subsystems, KAAS (KEGG Automatic Annotation Server), and the Carbohydrate-Active enZYmes (CAZy) database [53, 54]. For the KAAS- and CAZy-based analysis, concatenated protein fasta sequences of each MAG were uploaded to the KAAS and dbCAN webservers using the metagenome setting for KAAS (parameters: GHOSTX, genes dataset, SBH assignment method) and default settings for dbCAN.

Additionally, we searched for key metabolic genes using custom blast and hmmer databases using previously defined thresholds [23]. Therefore, we manually curated a reference blastp database including metabolic genes by searching the KEGG and NCBI databases for pathways and corresponding genes of interest. This reference database was screened against the concatenated protein fasta sequences from the MAGs using blastp (e-value threshold of 1e−20) [55]. Additionally, we utilized a published hmmer database using hmmsearch and custom bit score thresholds [23]. Hydrogenases were extracted from the genomes using hmmsearch (e-value cut-off of 1e−20), and hits were confirmed using a web-based search using the hydrogenase classifier HydDB [56]. Other positive hits of the blast or hmmer search were manually confirmed using a NCBI-based protein blast search [55]. The MAGs on average contained 1285 (archaea) to 2043 (bacteria) protein-coding genes, and of these, ~ 73% could be functionally assigned (Additional file 1: Table S3).

The core marker genes for processes shown in Figs. 1 and 2 as well as Additional file 1: Figures S3, S4, and S5 are listed in Additional file 1: Table S4. This table includes the information on the used databases as well as thresholds used for the functional annotation. Thresholds for the hmmer search were used as previously described [23]. Key enzymes and subunits were identified using hmmer searches, protein blast databases, and the KAAS and IMG/M systems. General pathway searches using the KAAS and IMG/M systems were used to confirm the presence of corresponding metabolic pathways in each of the bins. Functional processes were only considered if key enzymes/subunits were identified in multiple databases and when > 50% of the corresponding pathway components were detected in a genome.

### Phylogenetic analyses

Phylosift was used to extract marker genes for the phylogenetic placement of the assembled metagenomic bins [57]. These marker genes consist of up to 15 syntenic ribosomal protein genes that have been demonstrated to undergo limited lateral gene transfer (rpL2, 3, 4, 5, 6, 14, 15, 18, 22, 24 and rpS3, 8, 10, 17, 19) [58]. This gene set was derived from a reference database as detailed in [59]. To confirm the placement of the Candidatus Stahlbacteria as a separate phylum, we extracted up to 37 marker genes included in Phylosift for a more robust phylogenetic placement. These single-copy protein-coding markers include rpS2, rpS3, rpS5, rpS7, rpS8, rpS9, rpS10, rpS11, rpS12, rpS13, rpS15P, rpS17, rps19, rpL1, rpL2, rpL3, rpL4, rpL5, rpL6, rpL11, rpL13, rpL14b, rpL15, rpL16, rpL18P, rpL22, rpL24 rpL25, rpL29, IF-2, phenylalanyl-tRNA synthetase alpha and beta subunit, tRNA pseudouridine synthase B, porphobilinogen deaminase and the ribonuclease HII. To search for ribosomal protein sequences, all MAGs (fasta files) were used as an input in Phylosift, which was used with default parameters. Moreover, we included sequences from bacterial reference strains for phylogenetic analyses [60]. Amino acid alignments of the individual ribosomal protein genes were generated using MAFFT and manually curated [61]. For the extended phylogeny using 37 marker genes, we used the concatenated protein alignment provided by Phylosift. Afterwards, the curated alignments of the ribosomal proteins were concatenated for further phylogenetic analyses. For the ribosomal protein alignments, phylogenetic trees were generated using a maximum likelihood-based approach using RAxML (rate distribution models: PROTGAMMA, AA substitution model LG, rapid bootstrap analysis with 1000 replicates) or IQ-TREE (version 1.4.3, automatic model selection using jModelTest, ProtTest; ultrafast bootstrap with 1000 replicates) [62, 63].

## Additional file

Additional file 1: Supplementary Tables S1-S5 and Figures S1-S10. Tables S3 and S4 are provided separately. **Table S3.** Summary statistics of assembled archaeal and bacterial MAGs. Metagenome-assembled genomes (MAGs) assembled from dive 4484 and 4572. Providing information about the taxonomic affiliation (ribosomal protein phylogeny), completeness (CheckM) and genome statistics. The completeness lists the CheckM results, including the number of evaluated markers, the number of times a marker was found within a respective genome (numbers ranging from 0 to 5+) and the degrees of completeness, contamination, and heterogeneity. Summary statistics include the GC content (%), genome length (Mb), average coverage (based on read coverage of the individual contigs), number of called genes and average gene length (bp). **Table S4.** Functional analysis of assembled archaeal and bacterial MAGs. Functional gene analysis of individual archaeal and bacterial MAGs recovered from dive 4484 and 4572 based on HMMER, KAAS, JGI/M, and blastp analyses of marker genes for specific pathways involved in carbon (C), nitrogen (N), and sulfur (S) cycling as well as several additional pathways. Presence/absence of genes are listed as: Presence: >1 (red), Absence: 0 (no color). (ZIP 2404 kb)

## References

[1] Whitman WB, Coleman DC, Wiebe WJ (1998). Prokaryotes: the unseen majority. Proc Natl Acad Sci.

[2] Kallmeyer J, Pockalny R, Adhikari RR, Smith DC, D’Hondt S (2012). Global distribution of microbial abundance and biomass in subseafloor sediment. Proc Natl Acad Sci.

[3] Raven JA, Falkowski PG (1999). Oceanic sinks for atmospheric CO2. Plant Cell Environ.

[4] Masiello CA, Druffel ERM (1998). Black carbon in deep-sea sediments. Science.

[5] Simoneit BRT, Mazurek MA, Brenner S, Crisp PT, Kaplan IR (1979). Organic geochemistry of recent sediments from Guaymas Basin, Gulf of California. Deep Sea Res.

[6] Teske A, Callaghan AV, LaRowe DE (2014). Biosphere frontiers of subsurface life in the sedimented hydrothermal system of Guaymas Basin. Front Microbiol.

[7] Einsele G, Gieskes JM, Curray J, Moore DM, Aguayo E, Aubry M-P (1980). Intrusion of basaltic sills into highly porous sediments, and resulting hydrothermal activity. Nature.

[8] Bazylinski DA, Farrington JW, Jannasch HW (1988). Hydrocarbons in surface sediments from a Guaymas Basin hydrothermal vent site. Org Geochem.

[9] Von Damm KL, Edmond JM, Measures CI, Grant B (1985). Chemistry of submarine hydrothermal solutions at Guaymas Basin, Gulf of California. Geochim Cosmochim Acta.

[10] Pearson A, Seewald JS, Eglinton TI (2005). Bacterial incorporation of relict carbon in the hydrothermal environment of Guaymas Basin. Geochim Cosmochim Acta.

[11] Biddle JF, Cardman Z, Mendlovitz H, Albert DB, Lloyd KG, Boetius A (2012). Anaerobic oxidation of methane at different temperature regimes in Guaymas Basin hydrothermal sediments. ISME J..

[12] McKay L, Klokman VW, Mendlovitz HP, LaRowe DE, Hoer DR, Albert D (2016). Thermal and geochemical influences on microbial biogeography in the hydrothermal sediments of Guaymas Basin, Gulf of California. Environ Microbiol Rep.

[13] Dowell F, Cardman Z, Dasarathy S, Kellermann MY, Lipp JS, Ruff SE (2016). Microbial communities in methane- and short chain alkane-rich hydrothermal sediments of Guaymas Basin. Front Microbiol.

[14] Knittel K, Boetius A (2009). Anaerobic oxidation of methane: progress with an unknown process. Annu Rev Microbiol.

[15] Holler T, Widdel F, Knittel K, Amann R, Kellermann MY, Hinrichs K-U (2011). Thermophilic anaerobic oxidation of methane by marine microbial consortia. ISME J..

[16] Wegener G, Krukenberg V, Riedel D, Tegetmeyer HE, Boetius A (2015). Intercellular wiring enables electron transfer between methanotrophic archaea and bacteria. Nature.

[17] Krukenberg V, Harding K, Richter M, Glöckner FO, Gruber-Vodicka HR, Adam B (2016). Candidatus Desulfofervidus auxilii, a hydrogenotrophic sulfate-reducing bacterium involved in the thermophilic anaerobic oxidation of methane. Environ Microbiol.

[18] Meyer S, Wegener G, Lloyd KG, Teske A, Boetius A, Ramette A (2013). Microbial habitat connectivity across spatial scales and hydrothermal temperature gradients at Guaymas Basin. Front Microbiol..

[19] McKay LJ, MacGregor BJ, Biddle JF, Albert DB, Mendlovitz HP, Hoer DR (2012). Spatial heterogeneity and underlying geochemistry of phylogenetically diverse orange and white Beggiatoa mats in Guaymas Basin hydrothermal sediments. Deep Sea Res.

[20] Lin Y-S, Heuer VB, Goldhammer T, Kellermann MY, Zabel M, Hinrichs K-U (2012). Towards constraining H2 concentration in subseafloor sediment: a proposal for combined analysis by two distinct approaches. Geochim Cosmochim Acta.

[21] Teske A, de Beer D, McKay LJ, Tivey MK, Biddle JF, Hoer D (2016). The Guaymas Basin hiking guide to hydrothermal mounds, chimneys, and microbial mats: complex seafloor expressions of subsurface hydrothermal circulation. Front Microbiol.

[22] Baker BJ, Lazar CS, Teske AP, Dick GJ (2015). Genomic resolution of linkages in carbon, nitrogen, and sulfur cycling among widespread estuary sediment bacteria. Microbiome.

[23] Anantharaman K, Brown CT, Hug LA, Sharon I, Castelle CJ, Probst AJ (2016). Thousands of microbial genomes shed light on interconnected biogeochemical processes in an aquifer system. Nat Commun.

[24] Peters JW (1999). Structure and mechanism of iron-only hydrogenases. Curr Opin Struct Biol.

[25] Bian X-Y, Mbadinga SM, Liu Y-F, Yang S-Z, Liu J-F, Ye R-Q (2015). Insights into the anaerobic biodegradation pathway of n-alkanes in oil reservoirs by detection of signature metabolites. Sci Rep.

[26] Rabus R, Boll M, Heider J, Meckenstock RU, Buckel W, Einsle O (2016). Anaerobic microbial degradation of hydrocarbons: from enzymatic reactions to the environment. J Mol Microbiol Biotechnol.

[27] Callaghan AV, Davidova IA, Savage-Ashlock K, Parisi VA, Gieg LM, Suflita JM (2010). Diversity of benzyl- and alkylsuccinate synthase genes in hydrocarbon-impacted environments and enrichment cultures. Environ Sci Technol.

[28] Lloyd KG, Lapham L, Teske A (2006). An anaerobic methane-oxidizing community of ANME-1b archaea in hypersaline Gulf of Mexico sediments. Appl Environ Microbiol.

[29] Hallam SJ, Putnam N, Preston CM, Detter JC, Rokhsar D, Richardson PM (2004). Reverse methanogenesis: testing the hypothesis with environmental genomics. Science.

[30] Scheller S, Goenrich M, Boecher R, Thauer RK, Jaun B (2010). The key nickel enzyme of methanogenesis catalyses the anaerobic oxidation of methane. Nature.

[31] Schreiber L, Holler T, Knittel K, Meyerdierks A, Amann R (2010). Identification of the dominant sulfate-reducing bacterial partner of anaerobic methanotrophs of the ANME-2 clade. Environ Microbiol.

[32] Green-Saxena A, Dekas AE, Dalleska NF, Orphan VJ (2014). Nitrate-based niche differentiation by distinct sulfate-reducing bacteria involved in the anaerobic oxidation of methane. ISME J.

[33] Laso-Pérez R, Wegener G, Knittel K, Widdel F, Harding KJ, Krukenberg V (2016). Thermophilic archaea activate butane via alkyl-coenzyme M formation. Nature.

[34] Shi L, Richardson DJ, Wang Z, Kerisit SN, Rosso KM, Zachara JM (2009). The roles of outer membrane cytochromes of Shewanella and Geobacter in extracellular electron transfer. Environ Microbiol Rep.

[35] Reguera G, McCarthy KD, Mehta T, Nicoll JS, Tuominen MT, Lovley DR (2005). Extracellular electron transfer via microbial nanowires. Nature.

[36] MacGregor BJ, Biddle JF, Harbort C, Matthysse AG, Teske A (2013). Sulfide oxidation, nitrate respiration, carbon acquisition, and electron transport pathways suggested by the draft genome of a single orange Guaymas Basin Beggiatoa (Cand. Maribeggiatoa) sp. filament. Mar. Genomics.

[37] Bowles MW, Nigro LM, Teske AP, Joye S (2012). Denitrification and environmental factors influencing nitrate removal in Guaymas Basin hydrothermally altered sediments. Aquat Microbiol.

[38] Kelley CA, Martens CS, Chanton JP (1990). Variations in sedimentary carbon remineralization rates in the White Oak River estuary, North Carolina. Limnol Oceanogr.

[39] Lloyd KG, Alperin MJ, Teske A (2011). Environmental evidence for net methane production and oxidation in putative ANaerobic MEthanotrophic (ANME) archaea. Environ Microbiol.

[40] Kimes N, Callaghan A, Aktas D, Smith W, Sunner J, Golding B (2013). Metagenomic analysis and metabolite profiling of deep–sea sediments from the Gulf of Mexico following the Deepwater Horizon oil spill. Front Microbiol.

[41] Kniemeyer O, Musat F, Sievert SM, Knittel K, Wilkes H, Blumenberg M (2007). Anaerobic oxidation of short-chain hydrocarbons by marine sulphate-reducing bacteria. Nature.

[42] Knittel K, Lösekann T, Boetius A, Kort R, Amann R (2005). Diversity and distribution of methanotrophic archaea at cold seeps. Appl Environ Microbiol.

[43] Evans PN, Parks DH, Chadwick GL, Robbins SJ, Orphan VJ, Golding SD (2015). Methane metabolism in the archaeal phylum Bathyarchaeota revealed by genome-centric metagenomics. Science.

[44] Martin M (2011). Cutadapt removes adapter sequences from high-throughput sequencing reads. EMBnet J.

[45] Joshi NA, Fass JN. Sickle: A sliding-window, adaptive, quality-based trimming tool for FastQ files (Version 1.33) [Software]. 2011. Available at https://github.com/najoshi/sickle.

[46] Peng Y, Leung HCM, Yiu SM, Chin FYL (2012). IDBA-UD: a de novo assembler for single-cell and metagenomic sequencing data with highly uneven depth. Bioinformatics.

[47] Dick GJ, Andersson AF, Baker BJ, Simmons SL, Thomas BC, Yelton AP (2009). Community-wide analysis of microbial genome sequence signatures. Genome Biol.

[48] Baker BJ (2013). Omic approaches in microbial ecology: charting the unknown. Microbe..

[49] Parks DH, Imelfort M, Skennerton CT, Hugenholtz P, Tyson GW (2015). CheckM: assessing the quality of microbial genomes recovered from isolates, single cells, and metagenomes. Genome Res.

[50] Li H. Aligning sequence reads, clone sequences and assembly contigs with BWA-MEM. arXiv:1303.3997v1 [q-bio.GN]. 2013.

[51] Aziz RK, Bartels D, Best AA, DeJongh M, Disz T, Edwards RA (2008). The RAST Server: rapid annotations using subsystems technology. BMC Genomics.

[52] Overbeek R, Olson R, Pusch GD, Olsen GJ, Davis JJ, Disz T (2014). The SEED and the Rapid Annotation of microbial genomes using Subsystems Technology (RAST). Nucleic Acids Res.

[53] Moriya Y, Itoh M, Okuda S, Yoshizawa AC, Kanehisa M (2007). KAAS: an automatic genome annotation and pathway reconstruction server. Nucleic Acids Res.

[54] Lombard V, Ramulu HG, Drula E, Coutinho PM, Henrissat B (2014). The carbohydrate-active enzymes database (CAZy) in 2013. Nucleic Acids Res.

[55] Altschul SF, Madden TL, Schäffer AA, Zhang J, Zhang Z, Miller W (1997). Gapped BLAST and PSI-BLAST: a new generation of protein database search programs. Nucleic Acids Res.

[56] Søndergaard D, Pedersen CNS, Greening C (2016). HydDB: A web tool for hydrogenase classification and analysis. Sci Rep..

[57] Darling AE, Jospin G, Lowe E, Matsen FA, Bik HM, Eisen JA (2014). PhyloSift: phylogenetic analysis of genomes and metagenomes. PeerJ.

[58] Sorek R, Zhu Y, Creevey CJ, Francino MP, Bork P, Rubin EM (2007). Genome-wide experimental determination of barriers to horizontal gene transfer. Science.

[59] Castelle CJ, Wrighton KC, Thomas BC, Hug LA, Brown CT, Wilkins MJ (2015). Genomic expansion of domain archaea highlights roles for organisms from new phyla in anaerobic carbon cycling. Curr Biol.

[60] Hug LA, Baker BJ, Anantharaman K, Brown CT, Probst AJ, Castelle CJ (2016). A new view of the tree of life. Nat Microbiol.

[61] Katoh K, Misawa K, Kuma K, Miyata T (2002). MAFFT: a novel method for rapid multiple sequence alignment based on fast Fourier transform. Nucleic Acids Res.

[62] Stamatakis A (2014). RAxML version 8: a tool for phylogenetic analysis and post-analysis of large phylogenies. Bioinformatics.

[63] Nguyen L-T, Schmidt HA, von Haeseler A, Minh BQ (2015). IQ-TREE: a fast and effective stochastic algorithm for estimating maximum-likelihood phylogenies. Mol Biol Evol.

