# Supplementary material for: Genomic insights into potential interdependencies in microbial hydrocarbon and nutrient cycling in hydrothermal sediments
Source: Microbiome. 2017 Aug 23;5:106. doi: 10.1186/s40168-017-0322-2 (PMC5569505; doi:10.1186/s40168-017-0322-2)
Supplement: Additional file 1: — Supplementary Tables S1-S5 and Figures S1-S10. Tables S3 and S4 are provided separately. Table S3. Summary statistics of assembled archaeal and bacterial MAGs. Metagenome-assembled genomes (MAGs) assembled from dive 4484 and 4572. Providing information about the taxonomic affiliation (ribosomal protein phylogeny), completeness (CheckM) and genome statistics. The completeness lists the CheckM results, including the number of evaluated markers, the number of times a marker was found within a respective genome (numbers ranging from 0 to 5+) and the degrees of completeness, contamination, and heterogeneity. Summary statistics include the GC content (%), genome length (Mb), average coverage (based on read coverage of the individual contigs), number of called genes and average gene length (bp). Table S4. Functional analysis of assembled archaeal and bacterial MAGs. Functional gene analysis of individual archaeal and bacterial MAGs recovered from dive 4484 and 4572 based on HMMER, KAAS, JGI/M, and blastp analyses of marker genes for specific pathways involved in carbon (C), nitrogen (N), and sulfur (S) cycling as well as several additional pathways. Presence/absence of genes are listed as: Presence: >1 (red), Absence: 0 (no color). (ZIP 2404 kb) [file 40168_2017_322_MOESM1_ESM.zip › Additional_File1.docx]

# Additional file 1: Supplementary Tables

**Table S1: Sampling information for dive 4484 and 4572.** Information about sampling time, location, depth, *Beggiatoa* mat coverage, temperature, sulfate and methane concentrations recorded for dive 4484 and 4572.

| **Dive** | **4484** | | **4572** | |
| --- | --- | --- | --- | --- |
| **Date** | 12/2008 | | 12/2009 | |
| **Coordinates** | 27 00.388N, 111 W24.56 | | 27 00.423 N, 111 24.477 W | |
| **Core #** | 1 | | 18 | |
| **Beggiatoa Mat** | white mat | | orange mat | |
| **Depth (cm)** | 0-1 | 3-4 | 0-3 | 12-15 |
| **Temperature (°C)** | ~20 | ~60 | ~50 | ~80 |
| **Sulfate (mM)** | ~25 | ~10 | ~25 | ~15 |
| **Methane (mM)** | ~2 | ~4 | ~1 | ~15 |

**Table S2: Assembly statistics for samples sequenced from dive 4484 and 4572.** The two depth profiles sampled during dive 4484 and 4572 were co-assembled and this table provides information about the generated contigs (> 5000 bp), genes called and annotated open reading frames (ORFs).

|  | **Assembly 4484** | | **Assembly 4572** | |
| --- | --- | --- | --- | --- |
|  | **Total number** | **Percent (%)** | **Total number** | **Percent (%)** |
| Nr. of sequences (> 5kb) | 31440 | - | 27048 | - |
| Nr. of bases | 299376147 | - | 291954024 | - |
| N50 | 9634 | - | 11623 | - |
| CRISPR Count | 217 | - | 179 | - |
| RNA genes | 5795 | 1.74% | 3863 | 1.24% |
| rRNA genes | 259 | 0.08% | 141 | 0.05% |
| 5S rRNA | 93 | 0.03% | 62 | 0.02% |
| 16S rRNA | 83 | 0.02% | 34 | 0.01% |
| 18S rRNA | 2 | 0.00% | 1 | 0.00% |
| 23S rRNA | 80 | 0.02% | 43 | 0.01% |
| 28S rRNA | 1 | 0.00% | 1 | 0.00% |
| tRNA genes | 5536 | 1.67% | 3722 | 1.19% |
| Protein coding genes | 326579 | 98.26% | 308609 | 98.76% |
| with Product Name | 205041 | 61.69% | 185302 | 59.30% |
| with COG | 223834 | 67.34% | 200329 | 64.11% |
| with Pfam | 216426 | 65.12% | 199671 | 63.90% |
| with KO | 142391 | 42.84% | 124428 | 39.82% |
| with Enzyme | 75418 | 22.69% | 66408 | 21.25% |
| with MetaCyc | 46760 | 14.07% | 39853 | 12.75% |
| with KEGG | 82484 | 24.82% | 71522 | 22.89% |
| COG Clusters | 4283 | 92.49% | 4331 | 93.52% |
| Pfam Clusters | 5074 | 31.14% | 5398 | 33.13% |

**Table S3: Summary statistics of assembled archaeal and bacterial MAGs.** Metagenome-assembled genomes (MAGs) assembled from dive 4484 and 4572. Providing information about the taxonomic affiliation (ribosomal protein phylogeny), completeness (CheckM) and genome statistics. The completeness lists the CheckM results, including the number of evaluated markers, the number of times a marker was found within a respective genome (numbers ranging from 0 to 5+) and the degrees of completeness, contamination and heterogeneity. Summary statistics include the GC content (%), genome length (Mb), average coverage (based on read coverage of the individual contigs), number of called genes and average gene length (bp).

<Provided as separate file>

**Table S4: Functional analysis of assembled archaeal and bacterial MAGs.** Functional gene analysis of individual archaeal and bacterial MAGs recovered from dive 4484 and 4572 based on HMMER, KAAS, JGI/M and blastp analyses of marker genes for specific pathways involved in carbon (C), nitrogen (N) and sulfur (S)-cycling as well as several additional pathways. Presence/Absence of genes are listed as: Presence: >1 (red), Absence: 0 (no color).

<Provided as separate file>

**Table S5: Statistical comparison of genes encoding for carbohydrate-degrading enzymes and peptidases among archaeal and bacterial MAGs.** P-values were calculated by comparing the number of carbohydrate-degrading enzymes (CAZy) and peptidases among archaeal (n = 34) and bacterial (n = 77) MAGs (Figure 3). *: P-value < 0.05 (non-parametric Mann–Whitney test, Bonferroni corrected). Normalized by the total number of archaeal and bacterial MAGs. Statistics were performed separate for the two functional categories.

# Supplementary Figures

**Figure S1: Metagenomic binning using Emergent Self-Organizing Maps (ESOM).** Assembled genomic bins for dive 4484 (a) and 4572 (b) represented in an ESOM map. Dots represent genomic sequence fragments with a length > 5,000 bp that were clustered according to their tetranucleotide frequency. The color-coding refers to individual metagenomic bins.

**Figure S2: Phylogenetic tree of assembled archaeal MAGs using the 16S rRNA gene sequence.** Maximum-likelihood-based phylogenetic tree of 16S rRNA genes extracted from archaeal MAGs from Guaymas Basin deep-sea sediments. Red: Archaeal MAGs assembled from GB metagenomes. Bootstrap values were generated using the ultrafast bootstrap method (automatic replication selection). Dots: Bootstrap values between 70-100%, sized proportionally.

**Figure S3: Phylogenetic tree of assembled bacterial MAGs using the transcription factor IF2.** Unrooted maximum-likelihood-based phylogenetic tree of the transcription factor IF2 extracted from MAGs assembled from GB deep-sea sediments. Bootstrap values were generated using the ultrafast bootstrap method (1,000 replications). Dots: Bootstrap values between 70-100%, sized proportionally. Bold: Bacterial GB MAGs identified using ribosomal proteins (see Figure 1). Bacterial MAGs that were not identified using the concatenated set of ribosomal proteins due to lack of sequences are shown in red. For reference a subset of MAGs shown in Figure 1 were included.

**Figure S4: Phylogenetic tree of assembled bacterial MAGs using the ribosomal protein L15.** Rooted maximum-likelihood-based phylogenetic tree of the ribosomal protein L15 extracted from MAGs assembled from Guaymas Basin deep-sea sediments. Bootstrap values were generated using the ultrafast bootstrap methods (1,000 replications). Dots: Bootstrap values between 70-100%, sized proportionally. Bold: Bacterial GB MAGs identified using ribosomal proteins (see Fig. 1). Bacterial MAGs that were not identified using the concatenated set of ribosomal proteins due to lack of sequences are shown in red. For reference a subset of MAGs shown in Figure 1 were included.

**Figure S5: Phylogenetic tree of assembled bacterial MAGs using the ribosomal protein S2.** Unrooted maximum-likelihood-based phylogenetic tree of the ribosomal protein S2 (RPS2) extracte4d from MAGs assembled from Guaymas Basin deep-sea sediments. Bootstrap values were generated using the ultrafast bootstrap methods (1,000 replications). Dots: Bootstrap value between 70-100%, sized proportionally. Bold: Bacterial GB MAGs identified using ribosomal proteins (see Fig 1). Bacterial MAGs that were not identified using the concatenated set of ribosomal proteins due to lack of sequences are shown in red. For reference a subset of MAGs shown in Figure 1 were included.

**Figure S6: Phylogenetic tree of MAG ex4572_27 using 37 concatenated marker genes.** Maximum-likelihood-based phylogenetic tree 37 elite marker genes recovered from MAG ex4572_27 assembled from Guaymas Basin deep-sea sediments (bold). Bootstrap values were generated using the ultrafast bootstrap methods (automatic replication selection).

**Figure S7: Phylogenetic tree of assembled MAGs from the Candidatus Stahlbacteria (WOR-2) using 37 concatenated marker genes.** Maximum-likelihood phylogenetic tree based on 37 marker genes recovered from Candidatus Stahlbacteria MAGs (ex4484_18, ex4484_100) assembled from Guaymas Basin deep-sea sediments (bold). Bootstrap values were generated using the ultrafast bootstrap method (automatic replication selection). Number in brackets: Number of reference genomes included in collapsed nodes.

**Figure S8: Relative abundance of assembled archaeal MAGs among the different sampling sites.** Relative distribution of archaeal MAGs across samples 4484 0-1 cm (red), 4484 3-4 cm (dark red), 4572 0-3 cm (grey) and 4572 12-15 cm (black). The relative abundance was determined by read mapping using BWA (Burrows-Wheeler Alignment tool) using default settings and normalizing against total library size.

**Figure S9: Relative abundance of assembled bacterial MAGs among the different sampling sites.** Relative distribution of bacterial MAGs across samples 4484 0-1 cm (red), 4484 3-4 cm (dark red), 4572 0-3 cm (grey) and 4572 12-15 cm (black). The relative abundance was determined by read mapping using BWA (Burrows-Wheeler Alignment tool) using default settings and normalizing against total library size.

**Figure S10: Total number of genes encoding for carbohydrate-degrading enzymes and peptidases detected in individual archaeal and bacterial MAGs** (a) Total number of carbohydrate-degrading enzymes (CAZy) and (b) peptidases encoded in individual archaeal and bacterial MAGs (total number of genes).
